# Supplementary material for: The interactome of intact mitochondria by cross-linking mass spectrometry provides evidence for coexisting respiratory supercomplexes
Source: Mol Cell Proteomics. 2017 Dec 8;17(2):216–32. doi: 10.1074/mcp.RA117.000470 (PMC5795388; doi:10.1074/mcp.RA117.000470)
Supplement: Supplemental Data [file supp_RA117.000470_133922_0_supp_23978_fzffwf.zip › spectra_annotation/mito_DR_spectra_annotation/254-1-2-1-4-1.pdf]

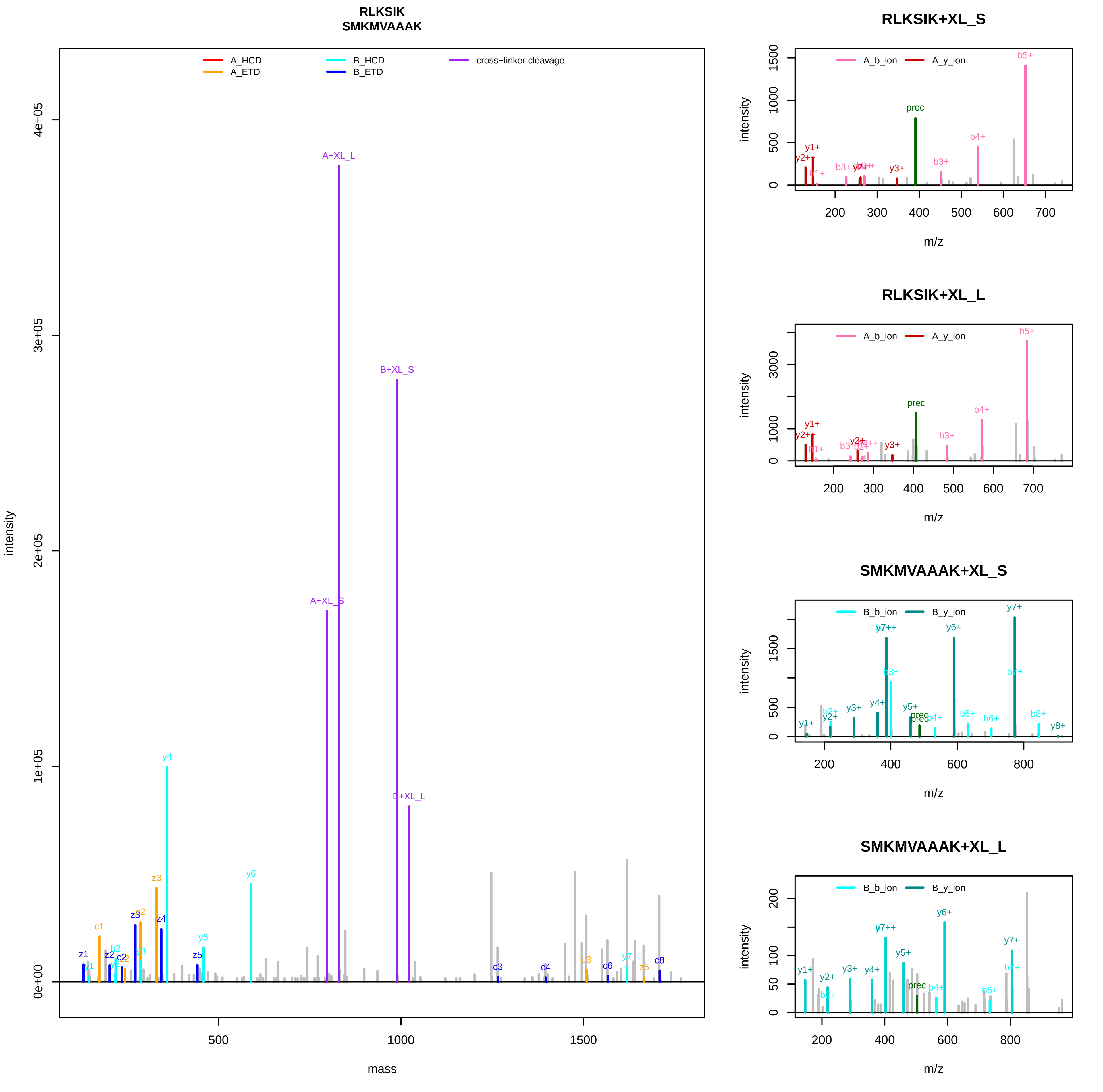
RLKSIK
SMKMVAAAK
A_HCD
A_ETD
B_HCD
B_ETD
cross−linker cleavage
intensity
mass
0e+00
1e+05
2e+05
3e+05
4e+05
500
1000
1500
A+XL_L
B+XL_S
A+XL_S
B+XL_L
y4
y6
y5
z3
z4
z5
c1
c3
c4
c6
c8
y7
z5
RLKSIK+XL_S
A_b_ion
A_y_ion
b5+
prec
b4+
b3+
y1+
y2+
b1+
y3+
m/z
RLKSIK+XL_L
SMKMVAAAK+XL_S
B_b_ion
B_y_ion
y7+
y7++
y6+
b3+
y4+
y5+
y3+
y2+
b2+
y1+
b4+
b5+
b6+
b7+
b8+
y8+
SMKMVAAAK+XL_L
